# Dermatomyositis: A Cancer Red Flag

**DOI:** 10.7759/cureus.32502

**Published:** 2022-12-14

**Authors:** Mariana Constante, Ana Rita Barradas, Ana Luísa Esteves, Sergio Pereira, Leandro Silva

**Affiliations:** 1 Internal Medicine Department, Egas Moniz Hospital, Lisbon, PRT; 2 Dermatology Department, Egas Moniz Hospital, Lisbon, PRT

**Keywords:** nxp-2 antibody, gottron papule, prostate cancer, paraneoplastic syndrome, dermatomyositis

## Abstract

Dermatomyositis is an inflammatory disease that affects muscle strength and causes skin manifestations. There is an increased incidence of cancer in patients with this diagnosis although the pathophysiology of this association is still not completely understood.

We report a case of a 65-year-old man who presented to the emergency department with proximal muscle weakness, weight loss, dysphagia, enlarged supraclavicular lymph nodes, an erythematous rash in the malar and supraciliary regions, and papules in the extensor metacarpophalangeal and interphalangeal joints. He had elevated creatine kinase and positive anti-nuclear matrix protein-2 autoantibodies. The skin and muscle biopsies performed confirmed the diagnosis of dermatomyositis. A thorough investigation seeking an associated condition was conducted and a prostate adenocarcinoma was diagnosed. The patient was treated with glucocorticoids and intravenous immune globulin with dysphagia and muscle weakness improvement and therefore allowing hospital discharge. He is currently undergoing oncologic treatment.

Myositis-specific antibodies have proved to be extremely useful in the diagnosis, prognosis, and management of patients with dermatomyositis. Various phenotypes of the disease can associate differently with a systemic condition (namely a malignant disease). This case illustrates a rare form of cancer presentation that every clinician, especially those who work in the emergency room or in primary care and therefore have immediate contact with many patients, must be able to recognize.

## Introduction

Dermatomyositis is an idiopathic inflammatory entity, more frequent in women, which is characterized by proximal skeletal muscle weakness (due to muscle inflammation) and typical skin manifestations [1].

Muscular weakness is present in 90% of the people diagnosed with dermatomyositis and is usually symmetric and proximal. The most frequently involved muscle groups are the deltoids and hip flexors. The symptoms are typically insidious and therefore patients tend to seek medical attention months after their onset. Muscle pain, atrophy, and stiffness are not common.

Skin manifestations are various and may be extremely evident or quite subtle. The most typical lesions are Gottron papules, which usually occur symmetrically in the metacarpophalangeal and interphalangeal joints of the hand. Other cutaneous findings include the Gottron’s sign (macules in extensor surfaces of joints in the elbows, knees, or ankles), heliotrope rash (similar to the one present in systemic lupus erythematosus but not sparing eyelids and nasolabial folds), facial erythema (like that of systemic lupus erythematosus), nailfold abnormalities and other rarer signs [2].

It is estimated that the incidence of cancer in patients with dermatomyositis is five to seven times higher than for the general population although the pathophysiology of this association is still incompletely understood [3].

## Case presentation

A 65-year-old male with a history of hypertension and type-2 diabetes presents to the emergency room because he had been unable to walk up the stairs for seven days. He described difficulty swallowing and muscle weakness in both legs that had started a couple of months before and was gradually becoming worse. He had lost around 30 kilograms in the previous six months.

**Figure 1:**
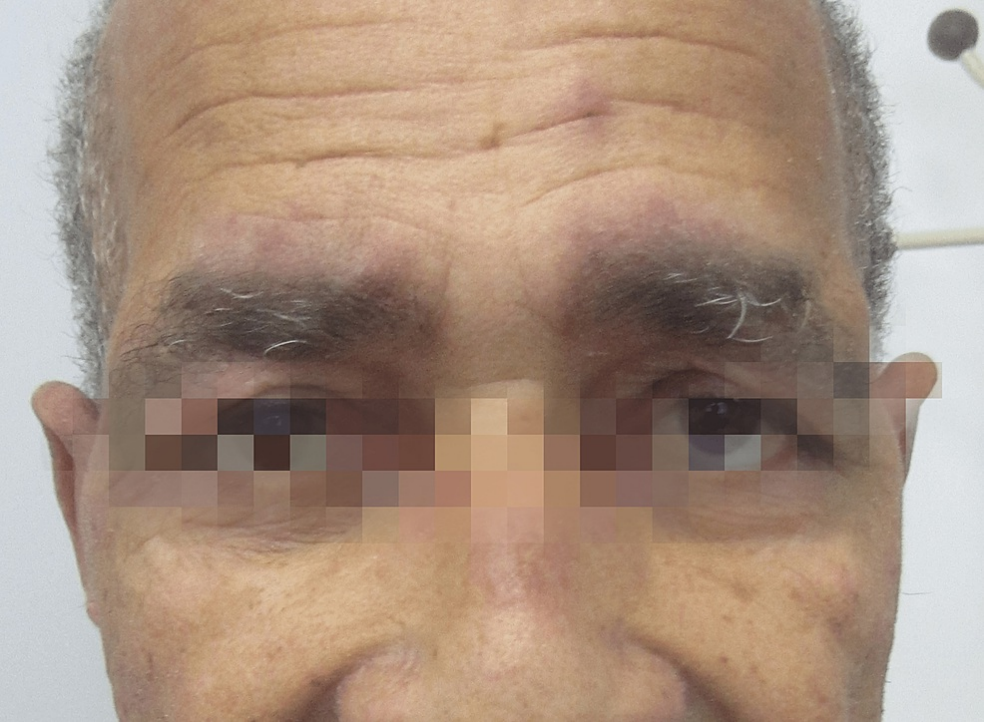
Erythematous rash in the malar and supraciliary regions

**Figure 2:**
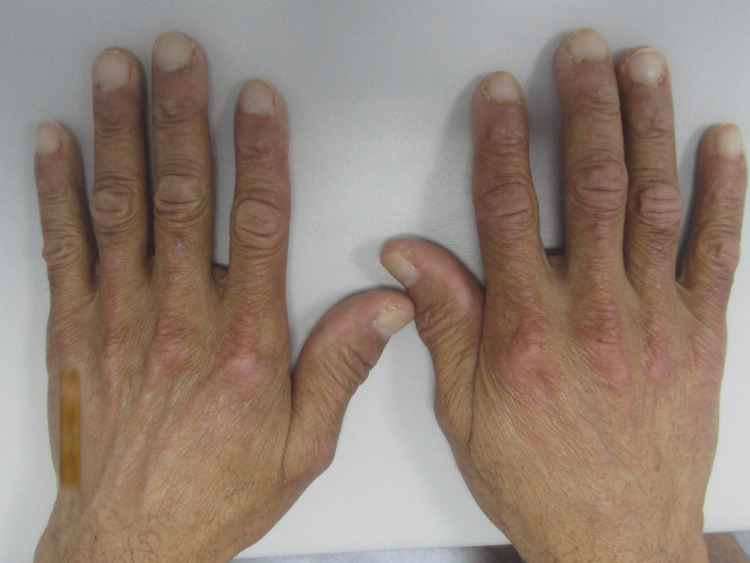
Gottron papules

At observation, he presented with two large, painful, hard, left supra-clavicular nodes and swelling of the left arm. The patient also had diminished muscle strength (3/5 in both legs). The skin showed an erythematous rash in the malar and supraciliary regions (Figure 1) and papules in the extensor metacarpophalangeal and interphalangeal joints - Gottron papules (Figure 2).

The blood work showed a significantly high creatine kinase of 1450 U/L (normal range <170 U/L) and positive anti-nuclear matrix protein-2 (NXP-2) autoantibodies. A skin biopsy was performed and its histologic exam was compatible with dermatomyositis; the muscle biopsy showed fiber atrophy and necrosis and the presence of mononuclear cells which the cytochemical stain marked as T lymphocytes. The hypothesis of dermatomyositis was confirmed after consulting with rheumatology, dermatology, and neurology specialists [4].

**Figure 3:**
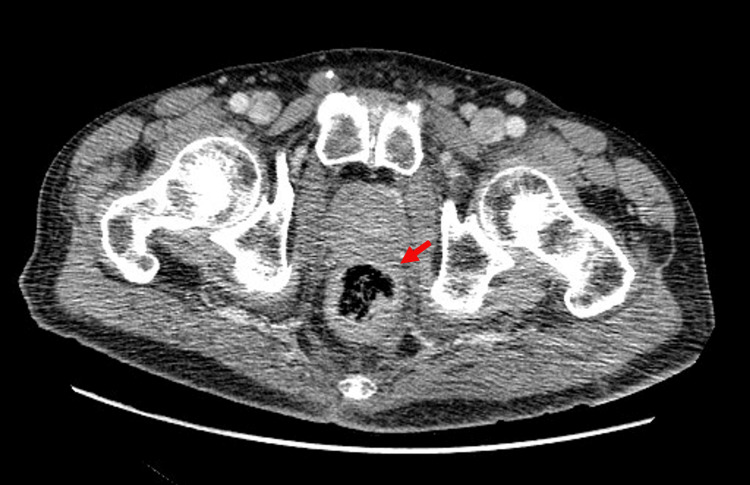
Pelvic computer tomography showing a thickened rectum with densified fat around the prostate.

In the pursuit of an associated condition, a biopsy of one of the enlarged supraclavicular lymph nodes was performed. It was positive for adenocarcinoma cells. Computer tomography showed a thickened rectum with densified fat around the prostate, a finding compatible with prostate malignancy, and multiple adenopathic conglomerates (in the iliac, lombo-aortic, inguinal, and supraclavicular groups). A urology consult was obtained, the digital rectal exam found a hard prostate and transperineal biopsy performed later revealed a multifocal acinar adenocarcinoma Gleason 9 (Figure 3).

The scintigraphy did not show bone metastases and the computer tomography did not show lung or liver metastases.

During the patient’s hospitalization, treatment with a high dose of prednisone was initiated immediately after the diagnosis. Since the clinical results were suboptimal and the patient maintained disabling muscle weakness and dysphagia, intravenous immune globulin (2 g/kg for five days) was added to the initial treatment with glucocorticoids. The symptoms remission was not complete, but hospital discharge was possible, and the patient continued physical rehabilitation at home [5].

The patient was referred to an oncology consultation and initiated prostate cancer treatment. Although great improvement of the symptoms was expected after control of the underlying condition (in this case, prostate cancer), this was not the case in our patient as after around one year of oncologic treatment he maintains muscle and skin manifestations of dermatomyositis.

## Discussion

Myositis’ specific antibodies have been studied in recent years and have proved to be extremely useful in the diagnosis, prognosis, and management of patients with dermatomyositis. On account of their high specificity, these antibodies may be very useful to establish the diagnosis. Furthermore, they define various dermatomyositis phenotypes and can indicate probability of association with a systemic condition (namely, a malign disease).

The specific antibody identified in the patient (NXP-2) is very rare in adults (present in between 25% and 18% of all cases of dermatomyositis) and is associated with greater muscle weakness, muscle atrophy, dysphagia, and significant functional impairment. The association between the NXP-2 antibody and the risk of malignancy is controverse but recent studies suggest it and therefore a thorough investigation is mandatory in this subgroup [6].

Our patient had other clinical elements that were deemed essential for specific exams to diagnose a possible underlying malignancy. This form of cancer presentation is rare and may be subtle but every clinician, especially those who work in the emergency room or in primary care and therefore have immediate contact with many patients, must be able to recognize these important signs.

## Conclusions

Dermatomyositis is a rare inflammatory condition that may present with obvious or subtle clinical signs and there is an association between dermatomyositis and malignancy, although the mechanisms behind it are not yet clear. Specific antibodies may be found in patients with dermatomyositis and are useful in the diagnosis, prognosis, and management of those patients.

The current case involved a multitude of medical resources, including diagnostic tests and the collaboration of several clinicians, namely, neurologists, dermatologists, rheumatologists, urologists, radiologists, pathologists, physiatrists, and oncologists. The internist must be, at all times, the captain who guides the ship towards the diagnosis (obvious or challenging) and who is able to recognize, even at a distance, a cancer red flag.
